# Age Moderates the Effect of Obesity on Mortality Risk in Critically Ill Patients With COVID-19: A Nationwide Observational Cohort Study*

**DOI:** 10.1097/CCM.0000000000005788

**Published:** 2023-02-10

**Authors:** Corstiaan A. den Uil, Fabian Termorshuizen, Wim J. R. Rietdijk, Roos S. G. Sablerolles, Hugo P. M. van der Kuy, Lenneke E. M. Haas, Peter H. J. van der Voort, Dylan W. de Lange, Peter Pickkers, Nicolette F. de Keizer, the Dutch COVID-19 Research Consortium

**Affiliations:** 1 Department of Intensive Care, Maasstad Hospital, Rotterdam, The Netherlands.; 2 Department of Intensive Care, Erasmus MC, University Medical Center, Rotterdam, The Netherlands.; 3 Department of Cardiology, Erasmus MC, University Medical Center, Rotterdam, The Netherlands.; 4 National Intensive Care Evaluation (NICE) Foundation, Amsterdam, The Netherlands.; 5 Department of Medical Informatics, Amsterdam UMC, Amsterdam Public Health research institute, University of Amsterdam, Amsterdam, The Netherlands.; 6 Department of Hospital Pharmacy, Erasmus MC, University Medical Center, Rotterdam, The Netherlands.; 7 Department of Internal Medicine, Erasmus MC, University Medical Center, Rotterdam, The Netherlands.; 8 Department of Intensive Care, Diakonessenhuis, Utrecht, The Netherlands.; 9 Department of Intensive Care, University Medical Center Groningen, Groningen, The Netherlands.; 10 Department of Intensive Care, Dutch Poisons Information Center (DPIC), University Medical Center, Utrecht University, Utrecht, The Netherlands.; 11 Department of Intensive Care, Radboud University Medical Center, Nijmegen, The Netherlands.

**Keywords:** age groups, COVID-19, critical illness, obesity, prognosis

## Abstract

A high body mass index (BMI) is associated with an unfavorable disease course in COVID-19, but not among those who require admission to the ICU. This has not been examined across different age groups. We examined whether age modifies the association between BMI and mortality among critically ill COVID-19 patients.

**DESIGN:**

An observational cohort study.

**SETTING:**

A nationwide registry analysis of critically ill patients with COVID-19 registered in the National Intensive Care Evaluation registry.

**PATIENTS:**

We included 15,701 critically ill patients with COVID-19 (10,768 males [68.6%] with median [interquartile range] age 64 yr [55–71 yr]), of whom 1,402 (8.9%) patients were less than 45 years.

**INTERVENTIONS:**

None.

**MEASUREMENTS AND MAIN RESULTS:**

In the total sample and after adjustment for age, gender, Acute Physiology and Chronic Health Evaluation IV, mechanical ventilation, and use of vasoactive drugs, we found that a BMI greater than or equal to 30 kg/m^2^ does not affect hospital mortality (adjusted odds ratio [OR_adj_] = 0.98; 95% CI, 0.90–1.06; *p* = 0.62). For patients less than 45 years old, but not for those greater than or equal to 45 years old, a BMI greater than or equal to 30 kg/m^2^ was associated with a lower hospital mortality (OR_adj_ = 0.59; 95% CI, 0.36–0.96; *p* = 0.03).

**CONCLUSIONS:**

A higher BMI may be favorably associated with a lower mortality among those less than 45 years old. This is in line with the so-called “obesity paradox” that was established for other groups of critically ill patients in broad age ranges. Further research is needed to understand this favorable association in young critically ill patients with COVID-19.

**KEY POINTS**

**Question**: What is the association between obesity and hospital mortality of patients admitted to the ICU with COVID-19 across different age strata?

**Findings**: In this observational national cohort study, we confirmed the lack of association between obesity and hospital mortality in the total sample. However, we found for the younger patient group (<45 yr) a favorable effect of a higher BMI on survival. The effect was both significant and clinically relevant: a 40% reduction in the odds of death.

**Meaning**: The obesity paradox may emerge in younger (<45 yr) critically ill patients with COVID-19.

The COVID-19 pandemic caused a surge of patients admitted to ICUs worldwide. In turn, the pandemic initiated research efforts focused on finding determinants, including body mass index (BMI), of outcome. Patients with higher BMI are more likely to experience a more severe course of COVID-19 (1). However, once admitted to the ICU, (severe) obesity does not clearly drive mortality as there is conflicting literature (2–4). Age and BMI in critically ill patients with COVID-19 are inversely correlated (5). Although there is much attention on BMI as a risk factor for mortality, the influence of this factor has not been examined across different age strata. Previous studies may have been underpowered as younger patients have been underrepresented in clinical studies (6, 7). We, therefore, aimed to describe and explore the association between obesity and hospital mortality of patients admitted to the ICU with COVID-19 across different age strata using updated Netherlands Intensive Care Evaluation (NICE) registry data.

## MATERIALS AND METHODS

### Data Collection and Study Population

We used patient data included in the national NICE quality registry, in which demographics, physiological and diagnostic data, ICU characteristics, and patient outcomes from all ICUs are registered (8). The data are prospectively collected. We included all adult patients (age >18 yr) who were admitted to the ICU between March 1, 2020, and January 1, 2022, with a confirmed COVID-19 infection. Compared with a previous study (2), we used the same registry data over the first half year of the pandemic, but now were able to extend the inclusion period up to 22 months. The Scientific Board of the NICE foundation (number 2021-01) a priori approved this study and its exploratory design, and the study was approved by the medical ethics committee of the Erasmus MC (MEC 2021-0646, August 26, 2021, title: “Does Obesity Interact With Age in Explaining Hospital Mortality in Critically Ill COVID-19 Patients? A Nationwide Registry Analysis”) that waived the need for informed consent. Procedures were followed in accordance with the ethical standards of the responsible MEC on human experimentation and with the Helsinki Declaration of 1975.

### Study Variables

For baseline characteristics, we included patient characteristics, comorbidities, admission characteristics including Acute Physiology and Chronic Health Evaluation (APACHE)-IV probability, complications during first 24 hours after ICU admission, and clinical outcomes. For patient characteristics, we included age, sex, and BMI and presented these as continuous variables (median [interquartile range (IQR)]) or as number (percentage), where appropriate. Length and weight were preferably measured but could be estimated. BMI was subdivided in several categories, and obesity was defined as a BMI greater than or equal to 30 kg/m^2^. Comorbidities are defined as chronic obstructive pulmonary disease/respiratory insufficiency, renal insufficiency (creatinine >177 μmol/L [2.0 mg/dL], or renal insufficiency in the medical history), liver cirrhosis, severe heart failure (NYHA class IV), malignancy including hematological, immune deficiency, and diabetes mellitus. In addition, we examined the number of comorbidities (i.e., 0, 1, and ≥2). We noted whether a patient was intubated and thus mechanically ventilated prior to or immediately after ICU admission. Events occurring the first 24 hours of ICU admission, like the start of mechanical ventilation in the first 24 hours, acute renal failure, and administration of vasoactive medication, were collected. Clinical outcomes are defined as inhospital mortality, ICU length of stay, and hospital length of stay.

### Study Endpoint

The endpoint is all-cause hospital mortality.

### Statistical Analysis

We described the study population in three age strata (i.e., <45, 45–65, and >65 yr), pragmatically chosen based on strata sizes and on previous studies (9). We analyzed the data in each age stratum according to the presence of obesity, and we compared survivors and nonsurvivors. Comparison of groups was done using a χ^2^ or Fisher exact test and with a Mann-Whitney *U* test or Kruskal Wallis test, when appropriate. We performed binary logistic regression models with hospital mortality as the outcome variable. We analyzed the associations between obesity (BMI ≥30 kg/m^2^) and hospital mortality stratified by age in three categories. This stratification was done by inclusion of terms for interaction of age × BMI. We built a multivariate model, where we included these terms for interaction and adjusted for APACHE-IV mortality probability in quintiles, age as continuous variable, gender, mechanical ventilation upon ICU admission, lowest Pao_2_/Fio_2_ ratio in quintiles, and the use of vasoactive drugs in the first 24 hours following ICU admission (2).

During the initial analysis, we found that the association between BMI and hospital mortality may be only present in patients under 45 years. For this reason, we decided to perform a post hoc analysis in this younger patient stratum. In this post hoc analysis, we explored whether one of the other study variables as confounders may explain the association between BMI and mortality. We examined the associations between obesity and hospital mortality controlled for several study variables in separate bivariate logistic regression models. (We used the following factors in these post-hoc regression models: gender, immuno-insufficiency, renal insufficiency, respiratory insufficiency, malignancy, cardiovascular disease, liver cirrhosis, at least one comorbidity, APACHE IV probability, diabetes mellitus, acute renal failure, mechanical ventilation [upon admission and in the first 24 hours], and the administration of vasoactive drugs.) For these regressions, we estimated the odds ratio (OR) and 95% CI. We examined statistical significance (*p* < 0.05) using a postestimation Wald test.

We performed sensitivity analyses for the univariate association between obesity and hospital mortality with differing BMI thresholds and age cutoffs. To check the assumption of linearity in our main multivariate model, we built an alternative multivariate model by entering age using refined categories (<45, 45–55, 55–60, 60–65, 65–70, 70–75, 75–80, and ≥80 yr) instead of a continuous variable in addition to age in broad categories. To check the initial results, we also built an alternative multivariable model where we included comorbidities and the terms for interaction of comorbidities × age and subdivided BMI in three categories (<25, 25–30, and >30 kg/m^2^). Regression diagnostics were performed to assess model fit (by eyeballing the calibration plot of 10%-categories of predicted versus observed mortality and by using the Hosmer-Lemeshow test and the deviance statistic) and to examine the potential for collinearity (through calculation of variance inflation factors [VIFs]).

To assess the impact of missing data on the results, we performed a sensitivity analysis using multiple imputation by chained equations of missing data for APACHE-IV probability, Pao_2_/Fio_2_, weight, length, and BMI. To assess the factor of time, we adjusted our main model by entering a categorized time variable, representing the series of COVID-19 waves and the periods in between. We also assessed the multivariate dose-response association between BMI and the risk for mortality from COVID-19 using BMI cutoffs of 25 and 30. Finally, to assess the impact of comorbidities on the association between BMI and mortality, we added the number of comorbidities to the main model and included the terms for interaction of age × BMI and age × number of comorbidities.

## RESULTS

We included 15,701 critically ill patients with COVID-19 (10,768 males, 68.6%, median age 64 [IQR, 55–71]). **Supplementary Table 1** (http://links.lww.com/CCM/H283) presents the characteristics of the sample and for each age stratum separately. The median APACHE-IV mortality probability at admission was 0.22 (IQR, 0.14–0.34). As for the younger patients (*n* = 1,402; 8.9%), the APACHE-IV probability was 0.10 (IQR, 0.07–0.16). The median BMI in the youngest patients was 30.5 (26.6–35.6), and this was significantly higher compared with the 45–65 years (29.4 [IQR, 26.3–33.3]) and greater than 65 year subgroups (27.8 [IQR, 25.1–31.1]). In general, the younger patients had less comorbidities compared with older patients. In **Supplementary Table 2** (http://links.lww.com/CCM/H283), we present the clinical characteristics for each age stratum according to BMI.

### Hospital Mortality Stratified by Age

Hospital mortality was 5.5%, 16.7%, and 42.1% (*p* < 0.001) for patients less than 45, 45–65, and greater than 65 years, respectively. The association of different levels of BMI with hospital mortality across the three age strata is listed in **Supplementary Table 3** (http://links.lww.com/CCM/H283). As for younger patients (<45 yr), we found differences in the number of comorbidities between survivors and nonsurvivors. Among the survivors, 81.2% had no comorbidities, whereas in the nonsurvivors, 61.0% had no comorbidities (*p* < 0.05).

### Hospital Mortality According to Obesity and Its Moderation by Age

**Table 1** presents the logistic regression analysis (including 15,321 out of the 15,701 = 97.6% of the total study sample) for the association between obesity and hospital mortality. In the total sample and without adjustment, we found that obesity is associated with a lower hospital mortality risk (OR, 0.74; 95% CI, 0.69–0.80; *p* < 0.001). After age stratification, we found a significant association between BMI greater than or equal to 30 kg/m^2^ and hospital mortality in patients less than 45 years (OR, 0.58; 95% CI, 0.36–0.93; *p* = 0.02). This association was not present in patients 45–65 years old and in those greater than 65 years old. The terms for interaction, however, did not reach the level of statistical significance (*p* = 0.12). The multivariate regression results showed that, in the total sample, the association disappeared, but the significant association between BMI greater than or equal to 30 kg/m^2^ and hospital mortality in patients less than 45 years (OR, 0.59; 95% CI, 0.36–0.96; *p* = 0.03) remained. This association was not found in patients 45–65 years old (OR, 1.05; 95% CI, 0.91–1.20) and in those greater than 65 years old (OR, 0.97; 95% CI, 0.87–1.08). In the multivariate model, the terms for interaction of age × BMI were borderline significant (*p* = 0.08) and, thus, became stronger. When the regression analysis was performed in those patients admitted to the ICU with a primary diagnosis of viral pneumonia (*n* = 14,425/15,321 [94.2%]), the OR for the association in patients less than 45 years remained similar in magnitude (OR, 0.61; 95% CI, 0.36–1.04), though the significance disappeared (*p* = 0.07, terms for interaction *p* = 0.10).

**TABLE 1.** The Binary Logistic Regression Analysis for the Univariate and Multivariate Association Between Obesity and Hospital Mortality

Model	Tested Category	Reference Category	Odds (Ref)	Probability (Ref)	OR	*p*	*p* for Interaction
Univariate model	Obesity (BMI ≥ 30)	BMI < 30, all ages	0.32 (0.43)	0.24 (0.30)	**0.74 (0.69–0.80**)	**< 0.001**	0.12
	Obesity (BMI ≥ 30; < 45 yr)	BMI < 30, <45 yr	0.04 (0.07)	0.04 (0.07)	**0.58 (0.36–0.93**)	**0.02**	
	Obesity (BMI ≥ 30; 45–65 yr)	BMI < 30, 45–65 yr	0.19 (0.20)	0.16 (0.17)	0.97 (0.85–1.10)	0.6	
	Obesity (BMI ≥ 30; > 65 yr)	BMI < 30, > 65 yr	0.68 (0.75)	0.41 (0.43)	0.91 (0.82–1.01)	0.08	
Multivariate model^a^	Obesity (BMI ≥ 30)	BMI < 30, all ages			0.98 (0.90–1.06)	0.62	0.08
	Obesity (BMI ≥ 30; < 45 yr)	BMI < 30, < 45 yr			**0.59 (0.36–0.96**)	**0.03**	
	Obesity (BMI ≥ 30; 45–65 yr)	BMI < 30, 45–65 yr			1.05 (0.91–1.20)	0.52	
	Obesity (BMI ≥ 30; > 65 yr)	BMI < 30, > 65 yr			0.97 (0.87–1.08)	0.55	

BMI = body mass index, OR = odds ratio.

a The multivariate model was adjusted for age, gender, Acute Physiology and Chronic Health Evaluation IV mortality probability (quintiles), mechanical ventilation at ICU admission, Pao_2_/Fio_2_ ratio (quintiles), and the use of vasoactive drugs.

Estimates are ORs and 95% CIs. *p* is based on the post hoc Wald test for significance of the estimates. Bold values are significant at 5% alpha level.

### Post Hoc Analysis

**Figure 1.**
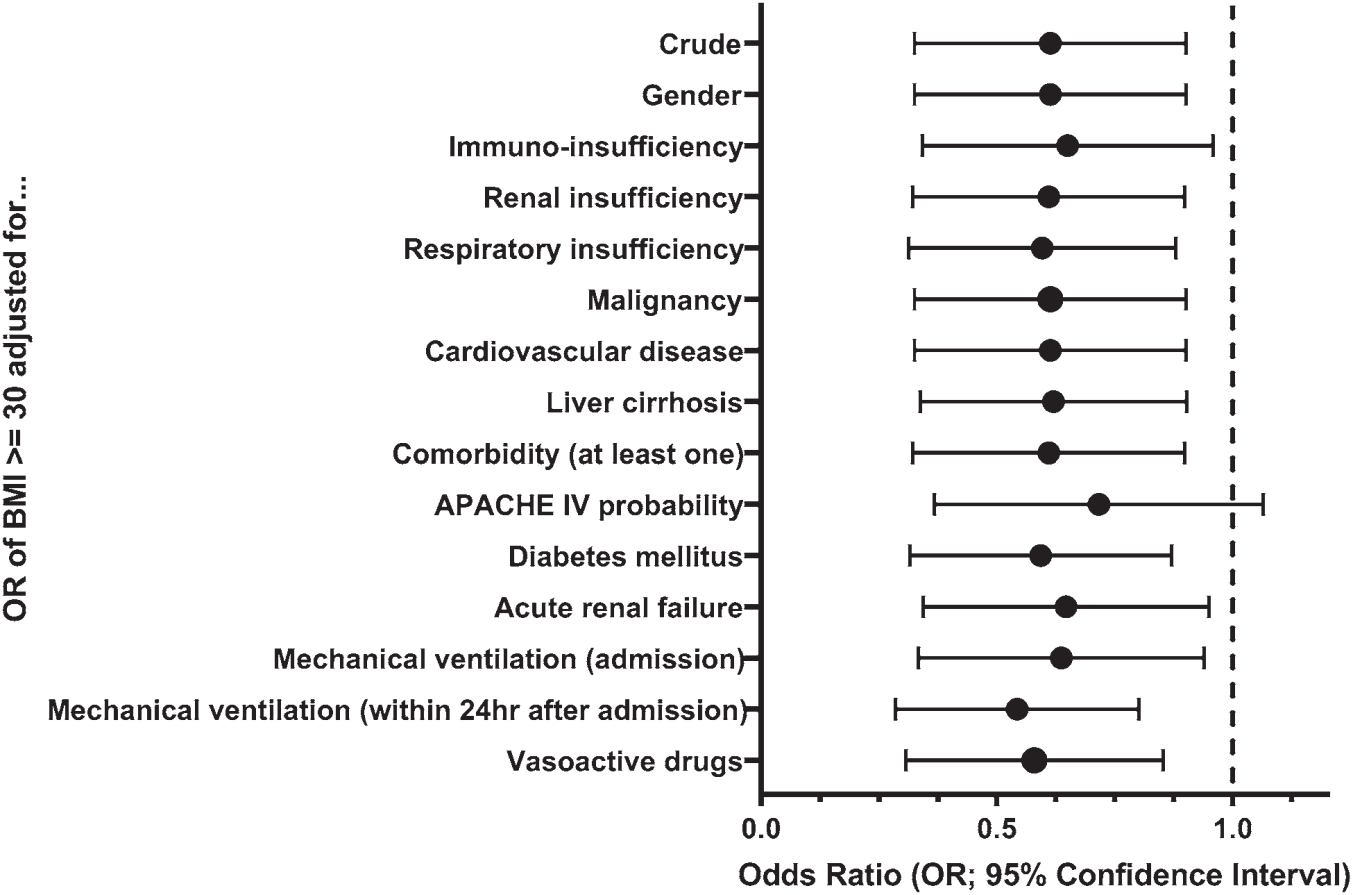
Post hoc analysis results: Odds ratios (ORs) and 95% CIs of the association between obesity and hospital mortality adjusted for several important clinical characteristics in younger (<45 yr) patients. APACHE = Acute Physiology and Chronic Health Evaluation, BMI = body mass index.

**Figure 1** presents the results of the post hoc analysis presenting the ORs for the association between obesity and hospital mortality when adjusting for several important clinical characteristics. The ORs remained similar in magnitude (OR between 0.50 and 0.65), though the significance disappeared when adjusting for APACHE-IV probability (*p* = 0.10). **Supplementary Table 4** (http://links.lww.com/CCM/H283) presents the full results of the post hoc regression analysis.

### Sensitivity Analyses

**Supplementary Tables 5** and **6** (http://links.lww.com/CCM/H283) demonstrate the univariate regression analyses with threshold at BMI = 25 kg/m^2^ and BMI = 35 kg/m^2^, respectively. These analyses confirm the lowest mortality risk associated with being overweight (OR = 0.50) or morbidly obese (OR = 0.77) in the youngest group. Although the effect seemed to weaken at a BMI threshold greater than or equal to 35 kg/m^2^, the interaction terms were borderline significant in both analyses. **Supplementary Tables 7** and **8** (http://links.lww.com/CCM/H283) show the univariate regression analyses using two different age cutoff values less than 40, 40–60, and greater than 60 years, and less than 55, 55–75, and greater than 75 years, respectively. This first analysis confirmed the lowest mortality risk, associated with obesity, in the youngest group. This effect disappeared in the second analysis where the youngest group was defined as less than 55 years. Entering age using refined categories into the model instead of the continuous variable for patients less than 45 years did not significantly change the results (data not shown). An alternative multivariable model where we included comorbidities and the terms for interaction of comorbidities × age, and subdivided BMI in three categories (<25, 25-30, and >30) showed again that the highest BMI category was associated with the lowest mortality for patients less than 45 years (*p* = 0.03, data not shown). Eyeballing our main model reflected very reasonable fit; however, the Hosmer-Lemeshow *p* value was significant, probably due to large patient numbers. Changing the main model by including age in refined categories did not improve model fit (H-L *p* value < 0.001). Assessing the model fit using the deviance statistic (in model with refined categories of age) and after entering age in refined categories (without age as continuous variable) experienced similar results (H-L *p* = 0.008 and *p* < 0.001, respectively). The fact that the model fit was not optimal when evaluated in terms of statistical significance is, therefore, not explained by the assumed linear relationship between log (odds) and age in the main analysis. There was no high correlation among the predictor variables (all VIFs <1.5), indicating no multicollinearity. There was no significant impact of missing data on the results (**Supplementary Table 9**, http://links.lww.com/CCM/H283). The results were similar when the main model was adjusted for the subsequent COVID-19 waves (data not shown). The dose-response association between BMI and the risk for mortality from COVID-19 using BMI cutoffs of 25 and 30 kg/m^2^ is demonstrated in **Supplementary Table 10** (http://links.lww.com/CCM/H283), where we demonstrate that for patients less than 45 years, “being obese” was associated with a lower hospital mortality than “being overweight,” and patients with BMI less than 25 kg/m^2^ had the highest mortality. **Supplementary Table 11** (http://links.lww.com/CCM/H283) demonstrates the regression analysis for the multivariate association between obesity and hospital mortality, after having added the number of comorbidities to the model, showing the ORs for all included variables.

## DISCUSSION

Although the literature is conflicting (3, 4), it was previously reported that the obesity paradox, that is a lower mortality in patients with a higher BMI, observed in various cohorts of critically ill patients, is not present in critically ill COVID-19 patients (2). Using updated NICE data, we confirmed the lack of association between obesity and hospital mortality in the total sample. However, after stratification by age, we found for the younger patient group (<45 yr) a favorable effect of a higher BMI on survival, indicating that, in this subgroup, the obesity paradox is indeed apparent. This paradox is not explained by age-dependent differences in APACHE-IV scores, gender, respiratory, or other parameters. The effect was both significant and clinically relevant: a 40% reduction in the odds of death. As the terms for interaction for age × BMI were borderline significant, our results suggest that obesity is an explanatory factor for lower hospital mortality among younger patients. This finding was further explored and confirmed using multiple post hoc and sensitivity analyses.

The question remains why a high BMI in younger patients results in a lower mortality. This may first be due to an unexplained biological mechanism, including a higher metabolic reserve in obese patients and differences in pulmonary mechanics and immunological aspects between obese and nonobese patients, especially in the young (10). Second, unmeasured confounders may have resulted in confounding bias (11). Confounding factors of the obesity-mortality relationship include unintended weight loss in the period preceding data collection, as well as data on premorbid physical wellness such as exercise tolerance, detailed preexisting heart disease, smoking, use of alcohol or drugs, socioeconomic status, and ethnicity (12–14). One may argue that particularly collider stratification bias may have partially explained our observations (15). Collider stratification bias may arise when one investigates a patient sample within a specific stratum, that is, for our study young patients with COVID-19 who required admission to the ICU (11, 16, 17). Younger obese patients have probably been less healthy or fit than in the hypothetical situation they would not have been obese. On the other hand, obese patients less than 45 years may just have been obese but may have suffered less from comorbidities. This is illustrated by the fact that young obese patients were less likely to have immune-insufficiency, and the highest tertile of APACHE-IV probability was less frequent in obese patients. We, therefore, performed the post hoc analysis and demonstrated that the association between BMI and mortality was consistent after adjusting for several important characteristics. It should be noted that the statistical significance disappeared when adjusting for the composite variable “APACHE-IV probability”; however, the consistent and large effect size (OR = 0.66) in addition to the multivariate regression results suggests that the effect or paradox is still present. Surge capacity issues may also have resulted in collider bias. Due to limited ICU capacity, one may expect a selection of patients with a higher BMI with COVID-19 but with less comorbidities and lower age to be admitted to the ICU, and this would plausibly translate into a better prognosis of these patients compared with patients with lower BMI but other prognostically less favorable reasons for ICU admission (13, 18, 19). We found an inverse relation between age and BMI; however, a higher BMI was not associated with the number of comorbidities in young patients. Finally, the same difference in percentages that are closer to 50% (as is the case with respect to mortality at higher ages) will lead to less extreme ORs, necessitating a larger sample size to reach the same statistical power. Thus, other factors such as high age and comorbidities may mask (“buffer”) the effect of high BMI at older age. Still, at young age, the largest difference in death rates between BMI greater than versus less than 30 was found. Thus, effect estimates both at a multiplicative and an additive scale suggest a survival benefit associated with a high BMI especially at young age.

Although we included all subsequent national ICU admissions from 22 months since the start of the pandemic, we acknowledge several methodological and other limitations. First, both the number of younger patients and death events in the young age group were relatively small and too limited to perform multiple subgroup analyses. Second, we mentioned methodological limitations including collider stratification bias above. Third, we assessed all-cause hospital mortality. It would have been informative to examine the cause of death between the subgroups (20), but this information is not available in the NICE registry, and there is unfortunately no record linkage possible with Statistics Netherlands. Fourth, our analyses might have suffered from some BMI group misallocation due to estimating rather than actually measuring height and weight. However, previous research demonstrated that either measurement or estimation of height and weight may not influence the association between BMI and mortality (21). Fifth, although we assessed the factor of time, treatment changes over time as well as (varying) shortage of ICU beds could have influenced the outcome in different age groups and BMI categories. These data were not available or could not be analyzed in detail. Future research should focus on a better understanding of the obesity paradox in critically ill patients with COVID-19 in an even larger database, particularly in younger patients. It is for future research relevant to examine different statistical methods, including machine learning approaches, more comprehensively (11). Further studies are also needed to investigate whether the obesity paradox observed for other critically ill patient groups is driven by the young.

## CONCLUSIONS

Overall, the obesity paradox is not present in critically ill patients with COVID-19, but we now report that it may emerge in younger (<45 yr) patients.

## Supplementary Material



## APPENDIX

The Dutch COVID-19 Research Consortium collaborators: M. S. Arbous, M. G. W. Barnas, D. P. Boer, R. J. Bosman, G. B. Brunnekreef, M. Th. de Bruin, M. J. de Graaff, R. M. de Jong, A. R. de Meijer, W. de Ruijter, R. de Waal, A. Dijkhuizen, D. A. Dongelmans, T. P. J. Dormans, A. Draisma, I. Drogt, B. J. W. Eikemans, P. W. G. Elbers, J. L. Epker, M. L. Erkamp, B. Festen-Spanjer, T. Frenzel, L. Georgieva, N. C. Gritters, I. Z. Hené, M. Hoeksema, J. W. M. Holtkamp, M. E. Hoogendoorn, C. J. G. M. Jacobs, I. T. A. Janssen, H. Kieft, M. P. Koetsier, T. J. J. Koning, H. Kreeftenberg, N. Kusadasi, J. A. Lens, J. G. Lutisan, D. J. Mehagnoul-Schipper, D. Moolenaar, F. Nooteboom, N. Postma, R. V. Pruijsten, D. Ramnarain, A. C. Reidinga, E. Rengers, A. A. Rijkeboer, T. Rijpstra, F. W. Rozendaal, R. M. Schnabel, V. M. Silderhuis, J. J. Spijkstra, P. E. Spronk, L. F. te Velde, L. C. Urlings-Strop, A. E. van den Berg, R. van den Berg, I. C. C. van der Horst, E. M. van Driel, L. van Gulik, F. M. van Iersel, M. van Lieshout, J. A. H. van Oers, E. R. van Slobbe-Bijlsma, M. van Tellingen, J. Vandeputte, D. P. Verbiest, D. J. Versluis, E. Verweij, M. Vrolijk-de Mos, and R. M. J. Wesselink.
