# Supplementary material for: Age Moderates the Effect of Obesity on Mortality Risk in Critically Ill Patients With COVID-19: A Nationwide Observational Cohort Study*
Source: Crit Care Med. 2023 Feb 10;51(4):484–91. doi: 10.1097/CCM.0000000000005788 (PMC10012838; doi:10.1097/CCM.0000000000005788)
Supplement: Supplementary file 1 [file ccm-51-484-s001.docx]

**Age moderates the effect of obesity on mortality risk in critically ill patients with COVID-19: A nationwide observational cohort study**

Corstiaan A. den Uil, M.D., Ph.D. ^a,b,c§^; Fabian Termorshuizen, Ph.D. ^d,e^; Wim J.R. Rietdijk, Ph.D. ^f^; Roos S.G. Sablerolles, M.D. ^f,g^; Hugo P.M. Van Der Kuy, Ph.D. ^f^; Lenneke E.M. Haas, M.D., Ph.D. ^h^; Peter H.J. van der Voort, M.D., Ph.D. ^I^; Dylan W. de Lange, M.D., PhD. ^j^; Peter Pickkers, M.D., Ph.D. ^k^; Nicolette F. de Keizer, Ph.D. ^d,e^; and the Dutch COVID-19 Research Consortium ^l^

****Supplementary digital content****

**Table of contents**

**Supplementary Table 1.** The characteristics of the study sample.

| Note: values are median (interquartile range) or number (percentage) for continuous and categorical variables, respectively. |
| --- |
| P-value is based on Fisher’s exact test and Kruskal Wallis test for categorical and continuous variables, respectively. |

**Supplementary Table 2.** Baseline characteristics according to the presence of obesity.

| Note: values are numbers (percentages) for categorical variables, respectively. P-values are based on chi-square tests. |
| --- |

**Supplementary Table 3.** Baseline characteristics comparing survivors and non-survivors.

| Note: values are median (interquartile range) or number (percentage) for continuous and categorical variables, respectively. |
| --- |
| P-value is based on Fisher's exact test and Mann Whitney U test for categorical and continuous variables, respectively. |

**Supplementary Table 4.** Full results of the post-hoc regression analysis in younger (<45 years) patients as presented in **Figure 1**.

**Supplementary Table 5.** The binary logistic regression analysis for the univariate association between obesity and hospital mortality using threshold BMI=25 kg/m^2^ instead of 30 kg/m^2^.

**Supplementary Table 6.** The binary logistic regression analysis for the univariate association between obesity and hospital mortality using threshold BMI=35 kg/m^2^ instead of 30 kg/m^2^.

**Supplementary Table 7.** The binary logistic regression analysis for the univariate association between obesity and hospital mortality using age cut-off values <40, 40-60, and >60 years instead of <45, 45-65, and >65 years.

**Supplementary Table 8.** The binary logistic regression analysis for the univariate association between obesity and hospital mortality using age cut-off values <55, 55-75, and >75 years instead of <45, 45-65, and >65 years.

**Supplementary Table 9.** The binary logistic regression analysis for the univariate and multivariate association between obesity and hospital mortality following multiple imputation of the original dataset.

**Supplementary Table 10.** The binary logistic regression analysis for the univariate and multivariate association between obesity and hospital mortality using 3 instead of 2 BMI categories.

**Supplementary Table 11.** The binary logistic regression analysis for the multivariate association between obesity and hospital mortality where the number of comorbidities was added to the main model.

**Supplementary Table 1.** The characteristics of the study sample.

| Characteristics | Category | Total sample | <45 years | 45-65 years | >65 years | p-value |
| --- | --- | --- | --- | --- | --- | --- |
|  |  | N= 15,701 | N= 1,402 (8.9) | N = 6,785 (43.2) | N = 7,514 (47.9) |  |
| Age | continuous | 64 (55-71) | 37 (31-41) | 57 (52-61) | 72 (68-75) |  |
|  | <20 years | 32 (0.2) | 32 (2.3) |  |  |  |
|  | 20-30 years | 239 (1.5) | 239 (17) |  |  |  |
|  | 30-40 years | 650 (4.1) | 650 (46.4) |  |  |  |
|  | 40-45 years | 481 (3.1) | 481 (34.3) |  |  |  |
|  | 45-50 years | 881 (5.6) |  | 881 (13) |  |  |
|  | 50-55 years | 1479 (9.4) |  | 1479 (21.8) |  |  |
|  | 55-60 years | 2028 (12.9) |  | 2028 (29.9) |  |  |
|  | 60-65 years | 2397 (15.3) |  | 2397 (35.3) |  |  |
|  | 65-70 years | 2679 (17.1) |  |  | 2679 (35.7) |  |
|  | 70-75 years | 2767 (17.6) |  |  | 2767 (36.8) |  |
|  | 75-80 years | 1611 (10.3) |  |  | 1611 (21.4) |  |
|  | 80-85 years | 402 (2.6) |  |  | 402 (5.4) |  |
|  | >85 years | 55 (0.4) |  |  | 55 (0.7) |  |
| Gender | Male | 10768 (68.6) | 792 (56.5) | 4633 (68.3) | 5343 (71.1) | <0.001 |
|  | Female | 4933 (31.4) | 610 (43.5) | 2152 (31.7) | 2171 (28.9) |  |
| BMI | Continuous | 28.7 (25.7-32.5) | 30.5 (26.6-35.6) | 29.4 (26.3-33.3) | 27.8 (25.1-31.1) | <0.001 |
|  | <18.5 | 72 (0.5) | 9 (0.6) | 24 (0.4) | 39 (0.5) | <0.001 |
|  | 18.5-<25 | 2976 (19) | 212 (15.1) | 1032 (15.2) | 1732 (23.1) |  |
|  | 25-<30 | 6170 (39.3) | 416 (29.7) | 2567 (37.8) | 3187 (42.4) |  |
|  | 30-<35 | 3815 (24.3) | 353 (25.2) | 1811 (26.7) | 1651 (22) |  |
|  | 35-<40 | 1547 (9.9) | 217 (15.5) | 766 (11.3) | 564 (7.5) |  |
|  | ≥40 | 776 (4.9) | 163 (11.6) | 446 (6.6) | 167 (2.2) |  |
|  | Unknown | 345 (2.2) | 32 (2.3) | 139 (2) | 174 (2.3) |  |
| Comorbidities | Immuno-insufficiency | 1475 (9.4) | 83 (5.9) | 609 (9) | 783 (10.4) | <0.001 |
|  | Renal insufficiency | 645 (4.1) | 21 (1.5) | 213 (3.1) | 411 (5.5) | <0.001 |
|  | Respiratory insufficiency | 1907 (12.1) | 76 (5.4) | 744 (11) | 1087 (14.5) | <0.001 |
|  | Malignancy | 395 (2.5) | 10 (0.7) | 141 (2.1) | 244 (3.2) | <0.001 |
|  | Cardiovascular disease | 242 (1.5) | 6 (0.4) | 53 (0.8) | 183 (2.4) | <0.001 |
|  | Liver cirrhosis | 66 (0.4) | 6 (0.4) | 26 (0.4) | 34 (0.5) | 0.8142 |
|  | Diabetes mellitus | 3510 (22.4) | 142 (10.1) | 1387 (20.4) | 1981 (26.4) | <0.001 |
| Number of comorbidities | 0 | 9394 (59.8) | 1123 (80.1) | 4318 (63.6) | 3953 (52.6) | <0.001 |
|  | 1 | 4691 (29.9) | 222 (15.8) | 1879 (27.7) | 2590 (34.5) |  |
|  | 2 | 1328 (8.5) | 50 (3.6) | 482 (7.1) | 796 (10.6) |  |
|  | 3 or 4 | 287 (1.8) | 7 (0.5) | 105 (1.5) | 175 (2.3) |  |
| APACHE IV probability | Continuous | 0.2 (0.1-0.3) | 0.1 (0.1-0.2) | 0.2 (0.1-0.3) | 0.3 (0.2-0.4) |  |
|  | quintile 1 (<20%) | 3086 (19.7) | 816 (58.2) | 1670 (24.6) | 600 (8) | <0.011 |
|  | quintile 2 (20-40%) | 3141 (20) | 333 (23.8) | 1925 (28.4) | 883 (11.8) |  |
|  | quintile 3 (40-60%) | 3144 (20) | 122 (8.7) | 1538 (22.7) | 1484 (19.7) |  |
|  | quintile 4 (60-80%) | 3144 (20) | 68 (4.9) | 936 (13.8) | 2140 (28.5) |  |
|  | quintile 5 (80-100%) | 3122 (19.9) | 59 (4.2) | 694 (10.2) | 2369 (31.5) |  |
|  | Unknown | 64 (0.4) | 4 (0.3) | 22 (0.3) | 38 (0.5) |  |
| Complications during first 24 hours at ICU | Acute renal failure | 1055 (6.7) | 40 (2.9) | 370 (5.5) | 645 (8.6) | <0.001 |
|  | Confirmed infection | 12514 (79.7) | 1067 (76.1) | 5473 (80.7) | 5974 (79.5) | <0.001 |
|  | Vasoactive medication | 7940 (50.6) | 503 (35.9) | 3419 (50.4) | 4018 (53.5) | <0.001 |
|  | Mechanical ventilation | 9683 (61.7) | 738 (52.6) | 4246 (62.6) | 4699 (62.5) | <0.001 |
| Clinical outcomes | pre-ICU hospital stay (days) | 1 (0-3) | 1 (0-3) | 1 (0-3) | 1 (0-3) | 0.002 |
|  | ICU length of stay | 12 (6-23) | 8 (4-14) | 12 (6-23) | 13 (6-25) | <0.001 |
|  | Hospital length of stay | 18 (11-32) | 13 (8-24) | 19 (12-33) | 19 (11-33) | <0.001 |
|  | Hospital mortality | 4372 (27.8) | 77 (5.5) | 1131 (16.7) | 3164 (42.1) | <0.001 |
| Note: values are median (interquartile range) or number (percentage) for continuous and categorical variables, respectively. | | | | | | |
| p-value is based on Fisher’s exact test and Kruskal Wallis test for categorical and continuous variables, respectively. | | | | | | |

**Supplementary Table 2.** Baseline characteristics according to the presence of obesity.

| Characteristics | Category | <45 years | | | 45-65 years | | | >65 years | | |
| --- | --- | --- | --- | --- | --- | --- | --- | --- | --- | --- |
|  |  | BMI <30 | BMI>=30 | p-value | BMI <30 | BMI>=30 | p-value | BMI <30 | BMI>=30 | p-value |
|  |  | N = 637 | N = 733 |  | N = 3,623 | N = 3,023 |  | N = 4,958 | N = 2,382 |  |
| Male |  | 387 (60.8) | 388 (52.9) | 0.004 | 2748 (75.9) | 1793 (59.3) | <0.001 | 3769 (76.0) | 1449 (60.8) | <0.001 |
| Female |  | 250 (39.3) | 345 (47.1) |  | 875 (24.2) | 1230 (40.7) |  | 1189 (24.0) | 933 (39.2) |  |
| Immuno-insufficiency |  | 46 (7.2) | 36 (4.9) | 0.09 | 372 (10.3) | 227 (7.5) | <0.001 | 555 (11.2) | 215 (9.0) | 0.005 |
| Renal insufficiency |  | 10 (1.6) | 10 (1.4) | 0.93 | 108 (3.0) | 101 (3.3) | 0.44 | 255 (5.1) | 144 (6.1) | 0.12 |
| Respiratory insufficiency |  | 24 (3.8) | 49 (6.7) | 0.02 | 361 (10.0) | 374 (12.4) | 0.002 | 667 (13.5) | 399 (16.8) | <0.001 |
| Malignancy |  | 6 (0.9) | 4 (0.6) | 0.59 | 101 (2.8) | 37 (1.2) | <0.001 | 176 (3.6) | 59 (2.5) | 0.02 |
| Cardiovascular disease |  | 4 (0.6) | 2 (0.3) | 0.56 | 27 (0.8) | 26 (0.9) | 0.7 | 116 (2.3) | 67 (2.8) | 0.26 |
| Liver cirrhosis |  | 5 (0.8) | 1 (0.1) | 0.16 | 8 (0.2) | 17 (0.6) | 0.04 | 22 (0.4) | 11 (0.5) | 1 |
| Diabetes mellitus |  | 61 (9.6) | 77 (10.5) | 0.63 | 630 (17.4) | 735 (24.3) | <0.001 | 1103 (22.3) | 833 (35.0) | <0.001 |
| Number of comorbidities | 0 | 514 (80.7) | 585 (79.8) | 0.82 | 2372 (65.5) | 1845 (61.0) | <0.001 | 2745 (55.4) | 1106 (46.4) | <0.001 |
|  | 1 | 96 (15.1) | 119 (16.2) |  | 953 (26.3) | 898 (29.7) |  | 1638 (33.0) | 903 (37.9) |  |
|  | 2 or more | 27 (4.2) | 29 (3.7) |  | 298 (8.2) | 280 (9.3) |  | 575 (11.6) | 373 (15.7) |  |
| Acute renal failure |  | 24 (3.8) | 14 (1.9) | 0.05 | 171 (4.7) | 185 (6.1) | 0.01 | 393 (7.9) | 230 (9.7) | 0.01 |
| Vasoactive medication |  | 226 (35.5) | 273 (37.2) | 0.54 | 1758 (48.5) | 1602 (53.0) | <0.001 | 2601 (52.5) | 1337 (56.1) | 0.003 |
| Mechanical ventilation (admission) |  | 169 (26.5) | 171 (23.3) | 0.19 | 1035 (28.6) | 859 (28.4) | 0.91 | 1459 (29.4) | 707 (29.7) | 0.84 |
| APACHE-IV probability | <33% | 487 (76.5) | 559 (76.5) | 0.005 | 1578 (43.6) | 1366 (45.3) | 0.06 | 776 (15.7) | 322 (13.5) | 0.001 |
|  | 33-66% | 91 (14.3) | 133 (18.2) |  | 1315 (36.4) | 1114 (36.9) |  | 1604 (32.5) | 868 (36.5) |  |
|  | >66% | 59 (9.3) | 39 (5.3) |  | 725 (20.0) | 537 (17.8) |  | 2559 (51.8) | 1189 (45.0) |  |
| Note: values are numbers (percentages) for categorical variables, respectively. P-values are based on chi-square tests. | | | | | | | | | | |

**Supplementary Table 3.** Baseline characteristics comparing survivors and non-survivors.

| Characteristics | Category | <45 years | | | 45-65 years | | | >65 years | | |
| --- | --- | --- | --- | --- | --- | --- | --- | --- | --- | --- |
|  |  | Survivors | Non-survivors | p-value | survivors | non-survivors | p-value | survivors | non-survivors | p-value |
|  |  | N = 1,325 | N = 77 |  | N = 5,654 | N = 1,131 |  | N = 4,350 | N = 3,164 |  |
| Age | <20 years | 30 (2.3) | 2 (2.6) | 0.12 |  |  |  |  |  |  |
|  | 20-30 years | 229 (17.3) | 10 (13.0) |  |  |  |  |  |  |  |
|  | 30-40 years | 621 (46.9) | 29 (37.7) |  |  |  |  |  |  |  |
|  | 40-45 years | 445 (33.6) | 36 (46.8) |  |  |  |  |  |  |  |
|  | 45-50 years |  |  |  | 804 (14.2) | 77 (6.8) | <0.001 |  |  |  |
|  | 50-55 years |  |  |  | 1296 (22.9) | 183 (16.2) |  |  |  |  |
|  | 55-60 years |  |  |  | 1714 (30.3) | 314 (27.8) |  |  |  |  |
|  | 60-65 years |  |  |  | 1840 (32.5) | 557 (49.3) |  |  |  |  |
|  | 65-70 years |  |  |  |  |  |  | 1805 (41.5) | 874 (27.6) | <0.001 |
|  | 70-75 years |  |  |  |  |  |  | 1582 (36.4) | 1185 (37.5) |  |
|  | 75-80 years |  |  |  |  |  |  | 788 (18.1) | 823 (26.0) |  |
|  | 80-85 years |  |  |  |  |  |  | 157 (3.6) | 245 (7.7) |  |
|  | >85 years |  |  |  |  |  |  | 18 (0.4) | 37 (1.2) |  |
| Gender | Male | 744 (56.2) | 48 (62.3) | 0.34 | 3835 (67.8) | 798 (70.6) | 0.08 | 2984 (68.6) | 2359 (74.6) | <0.001 |
|  | Female | 581 (43.9) | 29 (37.7) |  | 1819 (32.2) | 333 (29.4) |  | 1366 (31.4) | 805 (25.4) |  |
| BMI | <18.5 | 8 (0.6) | 1 (1.3) | 0.12 | 17 (0.3) | 7 (0.6) | 0.03 | 23 (0.5) | 16 (0.5) | 0.15 |
|  | 18.5-<25 | 193 (14.6) | 19 (24.7) |  | 859 (15.2) | 173 (15.3) |  | 954 (21.9) | 778 (24.6) |  |
|  | 25-<30 | 392 (29.6) | 24 (31.2) |  | 2141 (37.9) | 426 (37.7) |  | 1859 (42.7) | 1328 (44.0) |  |
|  | 30-<35 | 340 (25.7) | 13 (16.9) |  | 1547 (27.4) | 264 (23.3) |  | 983 (22.6) | 668 (21.1) |  |
|  | 35-<40 | 208 (15.7) | 9 (11.7) |  | 626 (11.1) | 140 (12.4) |  | 332 (7.6) | 232 (7.3) |  |
|  | ≥40 | 155 (11.7) | 8 (10.4) |  | 359 (6.4) | 87 (7.7) |  | 99 (2.3) | 68 (2.2) |  |
|  | Unknown | 29 (2.2) | 3 (3.9) |  | 105 (1.9) | 34 (3.0) |  | 100 (2.3) | 74 (2.3) |  |
| Comorbidities | Immuno-insufficiency | 70 (5.3) | 13 (16.9) | <0.001 | 417 (7.4) | 192 (17.0) | <0.001 | 365 (8.4) | 418 (13.2) | <0.001 |
|  | Renal insufficiency | 14 (1.1) | 7 (9.1) | <0.001 | 126 (2.2) | 87 (7.7) | <0.001 | 145 (3.3) | 266 (8.4) | <0.001 |
|  | Respiratory insufficiency | 69 (5.2) | 7 (9.1) | 0.23 | 566 (10.0) | 178 (15.7) | <0.001 | 539 (12.4) | 548 (17.3) | <0.001 |
|  | Malignancy | 9 (0.7) | 1 (1.3) | 1 | 76 (1.3) | 65 (5.8) | <0.001 | 106 (2.4) | 138 (4.4) | <0.001 |
|  | Cardiovascular disease | 5 (0.4) | 1 (1.3) | 0.76 | 36 (0.6) | 17 (1.5) | 0.0046 | 80 (1.8) | 103 (3.3) | <0.001 |
|  | Liver cirrhosis | 5 (0.4) | 1 (1.3) | 0.76 | 11 (0.2) | 15 (1.3) | <0.001 | 13 (0.3) | 21 (0.7) | 0.03 |
|  | Diabetes mellitus | 124 (9.4) | 18 (23.4) | <0.001 | 1095 (19.4) | 292 (25.8) | <0.001 | 1087 (25.0) | 894 (28.3) | 0.002 |
| Number of comorbidities | 0 | 1076 (81.2) | 47 (61.0) | <0.001 | 3762 (66.5) | 556 (49.2) | <0.001 | 2480 (57.0) | 1473 (46.6) | <0.001 |
|  | 1 | 207 (15.6) | 15 (19.5) |  | 1516 (26.8) | 363 (32.1) |  | 1464 (33.7) | 1126 (35.6) |  |
|  | 2 | 38 (2.9) | 12 (15.6) |  | 322 (5.7) | 160 (14.2) |  | 349 (8.0) | 447 (14.1) |  |
|  | 3 or 4 | 4 (0.3) | 3 (3.9) |  | 54 (1.0) | 51 (4.5) |  | 57 (1.3) | 118 (3.7) |  |
| APACHE IV probability | quintile 1 (<20%) | 799 (60.3) | 17 (22.1) | <0.001 | 1546 (27.3) | 124 (11.0) | <0.001 | 455 (10.5) | 145 (4.6) | <0.001 |
|  | quintile 2 (20-40%) | 315 (23.8) | 18 (23.4) |  | 1693 (29.9) | 232 (20.5) |  | 637 (14.6) | 246 (7.8) |  |
|  | quintile 3 (40-60%) | 111 (8.4) | 11 (14.3) |  | 1291 (22.8) | 247 (21.8) |  | 991 (22.8) | 493 (15.6) |  |
|  | quintile 4 (60-80%) | 56 (4.2) | 12 (15.6) |  | 711 (12.6) | 225 (19.9) |  | 1252 (28.8) | 888 (28.1) |  |
|  | quintile 5 (80-100%) | 40 (3.0) | 19 (24.7) |  | 396 (7.0) | 298 (26.4) |  | 989 (22.7) | 1380 (43.6) |  |
|  | Unknown | 4 (0.3) | 0 (0) |  | 17 (0.3) | 5 (0.4) |  | 26 (0.6) | 12 (0.4) |  |
| Complications during first 24 hours at ICU | Acute renal failure | 30 (2.3) | 10 (13.0) | <0.001 | 215 (3.8) | 155 (13.7) | <0.001 | 249 (5.72) | 396 (12.5) | <0.001 |
|  | Confirmed infection | 1015 (76.6) | 52 (67.5) | 0.09 | 4544 (80.4) | 929 (82.1) | 0.1815 | 3461 (79.6) | 2513 (79.4) | 0.91 |
|  | Vasoactive medication | 450 (34.0) | 53 (68.8) | <0.001 | 2718 (48.1) | 701 (62.0) | <0.001 | 2133 (49.0) | 1885 (59.6) | <0.001 |
|  | Mechanical ventilation | 674 (50.9) | 64 (83.1) | <0.001 | 3410 (60.3) | 836 (73.9) | <0.001 | 2537 (58.3) | 2162 (68.3) | <0.001 |
| Note: values are median (interquartile range) or number (percentage) for continuous and categorical variables, respectively. | | | | | | | | | | |
| p-value is based on Fisher's exact test and Mann Whitney U test for categorical and continuous variables, respectively. | | | | | | | | | | |

**Supplementary Table 4.** Full results of the post-hoc regression analysis in younger (<45 years) patients as presented in **Figure 1**.

| Regression model number; characteristic | | OR | Lower 95%CI | Upper 95%CI | Wald t-test | df | p-value |
| --- | --- | --- | --- | --- | --- | --- | --- |
| 1 | BMI <30 Crude | 1 | 1 | 1 |  |  |  |
|  | BMI >=30 Crude | 0.568 | 0.351 | 0.918 | 5.325 | 1 | 0.02102 |
| 2 | Gender: Male Crude | 1 | 1 | 1 |  |  |  |
|  | Gender: Female Crude | 0.77 | 0.473 | 1.255 | 1.096 | 1 | 0.29515 |
|  |  |  |  |  |  |  |  |
|  | BMI <30 Adjusted for gender | 1 | 1 | 1 |  |  |  |
|  | BMI >=30 Adjusted for gender | 0.568 | 0.351 | 0.918 | 5.325 | 1 | 0.02102 |
|  |  |  |  |  |  |  |  |
|  | Gender: Male Adjusted for BMI | 1 | 1 | 1 |  |  |  |
|  | Gender: Female Adjusted for BMI | 0.813 | 0.497 | 1.329 | 0.685 | 1 | 0.40787 |
| 3 | Immuno-insufficiency: No Crude | 1 | 1 | 1 |  |  |  |
|  | Immuno-insufficiency: Yes Crude | 4.016 | 2.073 | 7.778 | 16.992 | 1 | 4e-05 |
|  |  |  |  |  |  |  |  |
|  | BMI <30 Adjusted for Immuno-insufficiency | 1 | 1 | 1 |  |  |  |
|  | BMI >=30 Adjusted for Immuno-insufficiency | 0.601 | 0.37 | 0.975 | 4.246 | 1 | 0.03934 |
|  |  |  |  |  |  |  |  |
|  | Immuno-insufficiency: No Adjusted for BMI | 1 | 1 | 1 |  |  |  |
|  | Immuno-insufficiency: Yes Adjusted for BMI | 3.785 | 1.947 | 7.361 | 15.389 | 1 | 9e-05 |
| 4 | renal insufficiency: No Crude | 1 | 1 | 1 |  |  |  |
|  | renal insufficiency: Yes Crude | 9.993 | 3.826 | 26.101 | 22.082 | 1 | 0 |
|  |  |  |  |  |  |  |  |
|  | BMI <30 Adjusted for renal insufficiency | 1 | 1 | 1 |  |  |  |
|  | BMI >=30 Adjusted for renal insufficiency | 0.564 | 0.347 | 0.916 | 5.345 | 1 | 0.02078 |
|  |  |  |  |  |  |  |  |
|  | renal insufficiency: No Adjusted for BMI | 1 | 1 | 1 |  |  |  |
|  | renal insufficiency: Yes Adjusted for BMI | 10.053 | 3.838 | 26.331 | 22.066 | 1 | 0 |
| 5 | respiratory insufficiency: No Crude | 1 | 1 | 1 |  |  |  |
|  | respiratory insufficiency: Yes Crude | 1.837 | 0.809 | 4.174 | 2.112 | 1 | 0.14615 |
|  |  |  |  |  |  |  |  |
|  | BMI <30 Adjusted for respiratory insufficiency | 1 | 1 | 1 |  |  |  |
|  | BMI >=30 Adjusted for respiratory insufficiency | 0.553 | 0.341 | 0.896 | 5.783 | 1 | 0.01618 |
|  |  |  |  |  |  |  |  |
|  | respiratory insufficiency: No Adjusted for BMI | 1 | 1 | 1 |  |  |  |
|  | respiratory insufficiency: Yes Adjusted for BMI | 2.001 | 0.874 | 4.583 | 2.691 | 1 | 0.10092 |
| 6 | Malignancy: No Crude | 1 | 1 | 1 |  |  |  |
|  | Malignancy: Yes Crude | 2.063 | 0.255 | 16.699 | 0.461 | 1 | 0.49716 |
|  |  |  |  |  |  |  |  |
|  | BMI <30 Adjusted for Malignancy | 1 | 1 | 1 |  |  |  |
|  | BMI >=30 Adjusted for Malignancy | 0.571 | 0.353 | 0.923 | 5.227 | 1 | 0.02224 |
|  |  |  |  |  |  |  |  |
|  | Malignancy: No Adjusted for BMI | 1 | 1 | 1 |  |  |  |
|  | Malignancy: Yes Adjusted for BMI | 1.852 | 0.226 | 15.197 | 0.329 | 1 | 0.56625 |
| 7 | Cardiology: No Crude | 1 | 1 | 1 |  |  |  |
|  | Cardiology: Yes Crude | 4.989 | 0.534 | 46.597 | 1.988 | 1 | 0.15855 |
|  |  |  |  |  |  |  |  |
|  | BMI <30 Adjusted for Cardiology | 1 | 1 | 1 |  |  |  |
|  | BMI >=30 Adjusted for Cardiology | 0.571 | 0.353 | 0.924 | 5.206 | 1 | 0.02251 |
|  |  |  |  |  |  |  |  |
|  | Cardiology: No Adjusted for BMI | 1 | 1 | 1 |  |  |  |
|  | Cardiology: Yes Adjusted for BMI | 4.63 | 0.485 | 44.156 | 1.774 | 1 | 0.18289 |
| 8 | Liver cirrhosis: No Crude | 1 | 1 | 1 |  |  |  |
|  | Liver cirrhosis: Yes Crude | 3.39 | 0.388 | 29.621 | 1.219 | 1 | 0.26956 |
|  |  |  |  |  |  |  |  |
|  | BMI <30 Adjusted for Liver cirrhosis | 1 | 1 | 1 |  |  |  |
|  | BMI >=30 Adjusted for Liver cirrhosis | 0.575 | 0.355 | 0.931 | 5.072 | 1 | 0.02432 |
|  |  |  |  |  |  |  |  |
|  | Liver cirrhosis: No Adjusted for BMI | 1 | 1 | 1 |  |  |  |
|  | Liver cirrhosis: Yes Adjusted for BMI | 2.756 | 0.311 | 24.438 | 0.829 | 1 | 0.36256 |
| 9 | No comorbidity Crude | 1 | 1 | 1 |  |  |  |
|  | 1 comorbidity Crude | 1.753 | 0.955 | 3.219 | 40.914 | 2 | 0 |
|  | >=2 comorbidities Crude | 8.916 | 4.555 | 17.45 |  |  |  |
|  |  |  |  |  |  |  |  |
|  | BMI <30 Adjusted for comorbidity | 1 | 1 | 1 |  |  |  |
|  | BMI >=30 Adjusted for comorbidity | 0.564 | 0.346 | 0.921 | 5.247 | 1 | 0.02198 |
|  |  |  |  |  |  |  |  |
|  | No comorbidity Adjusted for BMI | 1 | 1 | 1 |  |  |  |
|  | 1 comorbidity Adjusted for BMI | 1.803 | 0.981 | 3.315 | 40.342 | 2 | 0 |
|  | >=2 comorbidities Adjusted for BMI | 8.934 | 4.536 | 17.593 |  |  |  |
| 10 | APACHE IV prob tertile 1 Crude | 1 | 1 | 1 |  |  |  |
|  | APACHE IV prob tertile 2 Crude | 2.693 | 1.451 | 4.998 | 68.528 | 2 | 0 |
|  | APACHE IV prob tertile 3 Crude | 11.947 | 6.64 | 21.493 |  |  |  |
|  |  |  |  |  |  |  |  |
|  | BMI <30 Adjusted for APACHE IV prob tertile | 1 | 1 | 1 |  |  |  |
|  | BMI >=30 Adjusted for APACHE IV prob tertile | 0.657 | 0.397 | 1.087 | 2.676 | 1 | 0.10187 |
|  |  |  |  |  |  |  |  |
|  | APACHE IV prob tertile 1 Adjusted for BMI | 1 | 1 | 1 |  |  |  |
|  | APACHE IV prob tertile 2 Adjusted for BMI | 2.739 | 1.475 | 5.086 | 64.496 | 2 | 0 |
|  | APACHE IV prob tertile 3 Adjusted for BMI | 11.24 | 6.226 | 20.292 |  |  |  |
| 11 | Diabetes: No Crude | 1 | 1 | 1 |  |  |  |
|  | Diabetes: Yes Crude | 3.056 | 1.735 | 5.384 | 14.951 | 1 | 0.00011 |
|  |  |  |  |  |  |  |  |
|  | BMI <30 Adjusted for diabetes | 1 | 1 | 1 |  |  |  |
|  | BMI >=30 Adjusted for diabetes | 0.546 | 0.336 | 0.886 | 5.992 | 1 | 0.01437 |
|  |  |  |  |  |  |  |  |
|  | Diabetes: No Adjusted for BMI | 1 | 1 | 1 |  |  |  |
|  | Diabetes: Yes Adjusted for BMI | 3.17 | 1.795 | 5.598 | 15.806 | 1 | 7e-05 |
| 12 | Acute renal failure: No Crude | 1 | 1 | 1 |  |  |  |
|  | Acute renal failure: Yes Crude | 5.894 | 2.666 | 13.033 | 19.199 | 1 | 1e-05 |
|  |  |  |  |  |  |  |  |
|  | BMI <30 Adjusted for acute renal failure | 1 | 1 | 1 |  |  |  |
|  | BMI >=30 Adjusted for acute renal failure | 0.596 | 0.366 | 0.968 | 4.377 | 1 | 0.03643 |
|  |  |  |  |  |  |  |  |
|  | Acute renal failure: No Adjusted for BMI | 1 | 1 | 1 |  |  |  |
|  | Acute renal failure: Yes Adjusted for BMI | 5.515 | 2.484 | 12.243 | 17.613 | 1 | 3e-05 |
| 13 | Mechanical ventilation at t0: No Crude | 1 | 1 | 1 |  |  |  |
|  | Mechanical ventilation at t0: Yes Crude | 2.618 | 1.624 | 4.221 | 15.607 | 1 | 8e-05 |
|  |  |  |  |  |  |  |  |
|  | BMI <30 Adjusted for Mechanical ventilation at t0 | 1 | 1 | 1 |  |  |  |
|  | BMI >=30 Adjusted for Mechanical ventilation at t0 | 0.591 | 0.364 | 0.958 | 4.551 | 1 | 0.0329 |
|  |  |  |  |  |  |  |  |
|  | Mechanical ventilation at t0: No Adjusted for BMI | 1 | 1 | 1 |  |  |  |
|  | Mechanical ventilation at t0: Yes Adjusted for BMI | 2.555 | 1.582 | 4.125 | 14.728 | 1 | 0.00012 |
| 14 | Mechanical ventilation at t24: No Crude | 1 | 1 | 1 |  |  |  |
|  | Mechanical ventilation at t24: Yes Crude | 4.345 | 2.362 | 7.993 | 22.324 | 1 | 0 |
|  |  |  |  |  |  |  |  |
|  | BMI <30 Adjusted for Mechanical ventilation at t24 | 1 | 1 | 1 |  |  |  |
|  | BMI >=30 Adjusted for Mechanical ventilation at t24 | 0.502 | 0.309 | 0.818 | 7.673 | 1 | 0.00561 |
|  |  |  |  |  |  |  |  |
|  | Mechanical ventilation at t24: No Adjusted for BMI | 1 | 1 | 1 |  |  |  |
|  | Mechanical ventilation at t24: Yes Adjusted for BMI | 4.639 | 2.514 | 8.559 | 24.102 | 1 | 0 |
| 15 | Vasoactive drugs: No Crude | 1 | 1 | 1 |  |  |  |
|  | Vasoactive drugs: Yes Crude | 4.197 | 2.528 | 6.965 | 30.782 | 1 | 0 |
|  |  |  |  |  |  |  |  |
|  | BMI <30 Adjusted for Vasoactive drugs | 1 | 1 | 1 |  |  |  |
|  | BMI >=30 Adjusted for Vasoactive drugs | 0.536 | 0.329 | 0.872 | 6.291 | 1 | 0.01214 |
|  |  |  |  |  |  |  |  |
|  | Vasoactive drugs at t24: No Adjusted for BMI | 1 | 1 | 1 |  |  |  |
|  | Vasoactive drugs at t24: Yes Adjusted for BMI | 4.307 | 2.589 | 7.162 | 31.653 | 1 | 0 |

**Supplementary Table 5.** The binary logistic regression analysis for the univariate association between obesity and hospital mortality using threshold BMI=25 kg/m^2^ instead of 30 kg/m^2^.

| Model | Tested category | Reference category | Odds (ref) | Probability (ref) | OR | p-value | | P-value for interaction | |
| --- | --- | --- | --- | --- | --- | --- | --- | --- | --- |
| Univariate model | Overweight (BMI >=25) | BMI <25, all ages | 0.36 (0.48) | 0.27 (0.33) | **0.75 (0.69-0.81)** | **<0.001** | |  | |
|  | Overweight (BMI >=25; <45 years) | BMI <25, <45 years | 0.05 (0.10) | 0.05 (0.09) | **0.50 (0.29-0.85)** | **0.01** | | 0.07 | |
|  | Overweight (BMI >=25; 45-65 years) | BMI <25, 45-65 years | 0.20 (0.21) | 0.16 (0.17) | 0.96 (0.80-1.14) | 0.61 | |  | |
|  | Overweight (BMI >=25; >65 years) | BMI <25, >65 years | 0.70 (0.81) | 0.41 (0.45) | **0.86 (0.77-0.96)** | **0.007** | |  | |
|  | | | | | | |  | |  |

Note: Estimates are Odds Ratios (ORs) and 95% Confidence Intervals (95% CIs). P-value is based upon the post-hoc Wald test for significance of the estimates. Bold values are significant at 5% alpha level.

**Supplementary Table 6.** The binary logistic regression analysis for the univariate association between obesity and hospital mortality using threshold BMI=35 kg/m^2^ instead of 30 kg/m^2^.

| Model | Tested category | Reference category | Odds (ref) | Probability (ref) | OR | p-value | | P-value for interaction | |
| --- | --- | --- | --- | --- | --- | --- | --- | --- | --- |
| Univariate model | Morbid obesity (BMI >=35) | BMI <35, all ages | 0.31 (0.40) | 0.23 (0.29) | **0.77 (0.69-0.85)** | **<0.001** | |  | |
|  | Morbid obesity (BMI >=35; <45 years) | BMI <35, <45 years | 0.05 (0.06) | 0.04 (0.06) | 0.77 (0.44-1.34) | 0.35 | | 0.06 | |
|  | Morbid obesity (BMI >=35; 45-65 years) | BMI <35, 45-65 years | 0.23 (0.19) | 0.19 (0.16) | **1.21 (1.03-1.42)** | **0.02** | |  | |
|  | Morbid obesity (BMI >=35; >65 years) | BMI <35, >65 years | 0.70 (0.73) | 0.41 (0.42) | 0.95 (0.82-1.11) | 0.54 | |  | |
|  | | | | | | |  | |  |

Note: Estimates are Odds Ratios (ORs) and 95% Confidence Intervals (95% CIs). P-value is based upon the post-hoc Wald test for significance of the estimates. Bold values are significant at 5% alpha level.

**Supplementary Table 7.** The binary logistic regression analysis for the univariate association between obesity and hospital mortality using age cut-off values <40, 40-60, and >60 years instead of <45, 45-65, and >65 years.

| Model | Tested category | Reference category | Odds (ref) | Probability (ref) | OR | p-value | | P-value for interaction | |
| --- | --- | --- | --- | --- | --- | --- | --- | --- | --- |
| Univariate model | Obesity (BMI >=30) | BMI <30, all ages | 0.32 (0.43) | 0.24 (0.30) | **0.74 (0.69-0.80)** | **<0.001** | |  | |
|  | Obesity (BMI >=30; <40 years) | BMI <30, <40 years | 0.03 (0.06) | 0.03 (0.05) | 0.61 (0.32-1.16) | 0.13 | | 0.16 | |
|  | Obesity (BMI >=30; 40-60 years) | BMI <30, 40-60 years | 0.14 (0.14) | 0.12 (0.12) | 1.02 (0.85-1.21) | 0.86 | |  | |
|  | Obesity (BMI >=30; >60 years) | BMI <30, >60 years | 0.55 (0.63) | 0.35 (0.39) | **0.87 (0.80-0.95)** | **0.002** | |  | |
|  | | | | | | |  | |  |

Note: Estimates are Odds Ratios (ORs) and 95% Confidence Intervals (95% CIs). P-value is based upon the post-hoc Wald test for significance of the estimates. Bold values are significant at 5% alpha level.

**Supplementary Table 8.** The binary logistic regression analysis for the univariate association between obesity and hospital mortality using age cut-off values <55, 55-75, and >75 years instead of <45, 45-65, and >65 years.

| Model | Tested category | Reference category | Odds (ref) | Probability (ref) | OR | p-value | | P-value for interaction | |
| --- | --- | --- | --- | --- | --- | --- | --- | --- | --- |
| Univariate model | Obesity (BMI >=30) | BMI <30, all ages | 0.32 (0.43) | 0.24 (0.30) | **0.74 (0.69-0.80)** | **<0.001** | |  | |
|  | Obesity (BMI >=30; <55 years) | BMI <30, <55 years | 0.10 (0.10) | 0.09 (0.09) | 1.01 (0.80-1.27) | 0.94 | | 0.50 | |
|  | Obesity (BMI >=30; 55-75 years) | BMI <30, 55-75 years | 0.40 (0.44) | 0.28 (0.30) | **0.91 (0.83-0.99)** | **0.04** | |  | |
|  | Obesity (BMI >=30; >75 years) | BMI <30, >75 years | 1.02 (1.21) | 0.50 (0.55) | 0.84 (0.69-1.02) | 0.08 | |  | |
|  | | | | | | |  | |  |

Note: Estimates are Odds Ratios (ORs) and 95% Confidence Intervals (95% CIs). P-value is based upon the post-hoc Wald test for significance of the estimates. Bold values are significant at 5% alpha level.

**Supplementary Table 9.** The binary logistic regression analysis for the univariate and multivariate association between obesity and hospital mortality following multiple imputation of the original dataset.

| Model | | Tested category | | Reference category | Odds (ref) | Probability (ref) | OR | p-value | | P-value for interaction | |
| --- | --- | --- | --- | --- | --- | --- | --- | --- | --- | --- | --- |
| Univariate model | | Obesity (BMI >=30) | | BMI <30, all ages | 0.32 (0.43) | 0.24 (0.30) | **0.75 (0.70-0.81)** | **<0.001** | |  | |
|  | | Obesity (BMI >=30; <45 years) | | BMI <30, <45 years | 0.04 (0.07) | 0.04 (0.07) | **0.60 (0.38-0.96)** | **0.03** | | 0.13 | |
|  | | Obesity (BMI >=30; 45-65 years) | | BMI <30, 45-65 years | 0.20 (0.20) | 0.16 (0.17) | 0.98 (0.86-1.12) | 0.76 | |  | |
|  | | Obesity (BMI >=30; >65 years) | | BMI <30, >65 years | 0.68 (0.74) | 0.40 (0.42) | 0.92 (0.83-1.01) | 0.08 | |  | |
| Multivariate model* | | Obesity (BMI >=30) | | BMI <30, all ages |  |  | 0.98 (0.91-1.07) | 0.68 | |  | |
|  | | Obesity (BMI >=30; <45 years) | | BMI <30, <45 years |  |  | **0.61 (0.38-0.98)** | **0.04** | | 0.08 | |
|  | | Obesity (BMI >=30; 45-65 years) | | BMI <30, 45-65 years |  |  | 1.05 (0.92-1.21) | 0.45 | |  | |
|  | | Obesity (BMI >=30; >65 years) | | BMI <30, >65 years |  |  | 0.97 (0.87-1.08) | 0.55 | |  | |
|  |  | |  | | | | | | |  | |
|  | | | | | | | | |  | |  |

Note: Estimates are Odds Ratios (ORs) and 95% Confidence Intervals (95% CIs). P-value is based upon the post-hoc Wald test for significance of the estimates. Bold values are significant at 5% alpha level.

*The multivariate model was adjusted for age, gender, APACHE-IV mortality probability (quintiles), mechanical ventilation at ICU admission, PaO_2_/FiO_2_ ratio (quintiles), and the use of vasoactive drugs.

**Supplementary Table 10.** The binary logistic regression analysis for the univariate and multivariate association between obesity and hospital mortality using 3 instead of 2 BMI categories.

| Model | Tested category | Reference category | Odds (ref) | Probability (ref) | OR | p-value | P-value for interaction |
| --- | --- | --- | --- | --- | --- | --- | --- |
| Univariate model | Overweight (BMI 25-30)  Obesity (BMI >= 30) | BMI <25, all ages | 0.40 (0.48)  0.32 (0.48) | 0.29 (0.33)  0.24 (0.33) | **0.84 (0.76-0.92)**  **0.66 (0.60-0.73)** | **<0.001** |  |
|  | Overweight (BMI 25-30; <45 years)  Obesity (BMI >=30; <45 years) | BMI <25, <45 years | 0.06 (0.10)  0.04 (0.10) | 0.06 (0.09)  0.04 (0.09) | **0.62 (0.33-1.14)**  **0.43 (0.24-0.77)** | **0.02** | 0.16 |
|  | Overweight (BMI 25-30; 45-65 years)  Obesity (BMI >=30; 45-65 years) | BMI <25, 45-65 years | 0.20 (0.21)  0.19 (0.21) | 0.17 (0.17)  0.16 (0.17) | 0.97 (0.80-1.17) 0.94 (0.78-1.14) | 0.82 |  |
|  | Overweight (BMI 25-30; >65 years)  Obesity (BMI >=30; >65 years) | BMI <25, >65 years | 0.71 (0.81)  0.68 (0.81) | 0.42 (0.45)  0.41 (0.45) | **0.88 (0.78-0.99)**  **0.84 (0.74-0.95)** | **0.02** |  |
| Multivariate model* | Overweight (BMI 25-30)  Obesity (BMI >= 30) | BMI <25, all ages |  |  | 0.93 (0.84-1.04)  0.94 (0.84-1.04) | 0.39 |  |
|  | Overweight (BMI 25-30; <45 years)  Obesity (BMI >=30; <45 years) | BMI <25, <45 years |  |  | **0.56 (0.30-1.05)**  **0.41 (0.23-0.75)** | **0.02** | 0.08 |
|  | Overweight (BMI 25-30; 45-65 years)  Obesity (BMI >=30; 45-65 years) | BMI <25, 45-65 years |  |  | 1.00 (0.82-1.22)  1.05 (0.86-1.27) | 0.81 |  |
|  | Overweight (BMI 25-30; >65 years)  Obesity (BMI >=30; >65 years) | BMI <25, >65 years |  |  | 0.93 (0.82-1.06)  0.93 (0.81-1.06) | 0.46 |  |

Note: Estimates are Odds Ratios (ORs) and 95% Confidence Intervals (95% CIs). P-value is based upon the post-hoc Wald test for significance of the estimates. Bold values are significant at 5% alpha level.

*The multivariate model was adjusted for age, gender, APACHE-IV mortality probability (quintiles), mechanical ventilation at ICU admission, PaO_2_/FiO_2_ ratio (quintiles), and the use of vasoactive drugs.

**Supplementary Table 11.** The binary logistic regression analysis for the multivariate association between obesity and hospital mortality where the number of comorbidities was added to the main model.

| Model | Tested category | Reference category | OR | p-value | P-value for interaction |
| --- | --- | --- | --- | --- | --- |
| Multivariate model* | Obesity (BMI >=30; <45 years) | BMI <30, <45 years | **0.59 (0.36-0.96)** | **0.03** | 0.10 |
|  | Obesity (BMI >=30; 45-65 years) | BMI <30, 45-65 years | 1.02 (0.88-1.17) | 0.84 |  |
|  | Obesity (BMI >=30; >65 years) | BMI <30, >65 years | 0.93 (0.84-1.04) | 0.21 |  |
|  |  |  |  |  |  |
|  | Comorbidities (1; <45 years) | Comorbidities (No; <45 years) | 1.48 (0.80-2.74) | **<0.001** | **0.006** |
|  | Comorbidities (≥2; <45 years) |  | **5.85 (2.90-11.8)** |  |  |
|  | Comorbidites (1; 45-65 years) | Comorbidities (No; 45-65 years) | **1.14 (1.18-1.60)** | **<0.001** |  |
|  | Comorbidities (≥2; 45-65 years) |  | **2.50 (2.04-3.08)** |  |  |
|  | Comorbidities (1; >65 years) | Comorbidities (No; >65 years) | **1.18 (1.06-1.32)** | **<0.001** |  |
|  | Comorbidities (≥2; >65 years) |  | **1.85 (1.58-2.15)** |  |  |
|  |  |  |  |  |  |
|  | APACHE IV probability 20-40% | APACHE IV probability <20% | **1.42 (1.20-1.67)** | **<0.001** |  |
|  | APACHE IV probability 40-60% |  | **1.77 (1.51-2.08)** |  |  |
|  | APACHE IV probability 60-80% |  | **2.26 (1.93-2.65)** |  |  |
|  | APACHE IV probability >80% |  | **3.93 (3.34-4.62)** |  |  |
|  |  |  |  |  |  |
|  | Age (continuous, years) |  | **1.06 (1.06-1.07)** | **<0.001** |  |
|  |  |  |  |  |  |
|  | Gender (female) | Gender (male) | **0.78 (0.72-0.86)** | **<0.001** |  |
|  |  |  |  |  |  |
|  | Mechanical ventilation at ICU admission | No mechanical ventilation | **1.31 (1.19-1.43)** | **<0.001** |  |
|  |  |  |  |  |  |
|  | Use of vasoactive drugs | No use of vasoactive drugs | **1.30 (1.19-1.41)** | **<0.001** |  |
|  |  |  |  |  |  |
|  | PaO_2_/FiO_2_ 20-<40% | PaO_2_/FiO_2_ <20% | **0.84 (0.73-0.97)** | **<0.001** |  |
|  | PaO_2_/FiO_2_ 40-<60% |  | **0.77 (0.67-0.89)** |  |  |
|  | PaO_2_/FiO_2_ 60-<80% |  | **0.74 (0.64-0.85)** |  |  |
|  | PaO_2_/FiO_2_ ≥80% |  | **0.70 (0.61-0.82)** |  |  |

Note: Estimates are Odds Ratios (ORs) and 95% Confidence Intervals (95% CIs). P-value is based upon the post-hoc Wald test for significance of the estimates. Bold values are significant at 5% alpha level.

*The multivariate model was adjusted for all covariates listed above, including the terms for interaction of age x BMI and age x number of comorbidities.
